# Negative Affect during a Collective (but Not an Individual) Task Is Associated with Holistic Attention in East Asian Cultural Context

**DOI:** 10.3389/fpsyg.2017.01283

**Published:** 2017-08-04

**Authors:** Hitoshi Tominaga, Yukiko Uchida, Yuri Miyamoto, Teruo Yamasaki

**Affiliations:** ^1^Graduate School of Human and Environmental Studies, Kyoto University Kyoto, Japan; ^2^Kokoro Research Center, Kyoto University Kyoto, Japan; ^3^Department of Psychology, University of Wisconsin-Madison Madison, WI, United States; ^4^Department of Psychology, Osaka Shoin Women's University Higashi-Osaka, Japan

**Keywords:** holistic attention, collective task, musical duet task, East Asian culture, Framed Line Test, social anxiety

## Abstract

Previous studies have suggested that individuals from East Asian cultures are more likely to show holistic attention—a pattern of attention that incorporates contextual information into focal stimuli—than individuals from North American cultures. Holistic attention is also prevalent in communities that require close cooperation. However, it is not yet known how cooperation is related to holistic attention. We theorized that holistic attention increases when people experience negative affect (e.g., worry, sadness, and frustration) during collective tasks (but not during individual tasks) because negative affect in social contexts signals the existence of potential threats to social harmony, thus indicating a need to restore social harmony. To examine this hypothesis, an experiment was conducted in which participants performed a musical duet either with another participant (a collective task requiring cooperation), or individually with a computer (an individual task). After the musical task, the Framed Line Task (FLT) was administered to examine their holistic attention. Participants also reported their emotional states both before and after the music task. Results suggested that negative affect in the collective task—but not the individual task—was positively correlated with a holistic pattern of attention. The function of negative affect in social contexts as motivation to restore relationships and how this enhances holistic attention is discussed. The moderating effect of social context on the link between negative affect and cognition is also discussed.

## Introduction

Previous studies on cultural cognition have shown that there are cultural differences in attention between people from North American and East Asian cultures (see Nisbett and Miyamoto, 2005 for a review). On the one hand, analytic attention, which is characterized by a focus on focal objects, independent of contextual information (Masuda and Nisbett, 2001; Kitayama et al., 2003), predominates in North American cultural contexts. On the other hand, holistic attention, which is characterized by attention across the whole field and to the relationship between focal objects and background information (Masuda and Nisbett, 2001; Kitayama et al., 2003; Chua et al., 2005), predominates in East Asian cultural contexts. Such cross-cultural differences have been demonstrated across a variety of tasks and methods, including eye-tracking (Chua et al., 2005) and fMRI studies (Hedden et al., 2008). In this study, we propose that holistic attention (vs. analytic attention) in the Japanese context is fostered by collective works, especially during tasks in which people experience negative affect related to social concerns, as negative affect in such contexts may signal potential threats to social harmony that motivate strategies to restore it.

### Culture and attention: the cultural task of collective work

As evidence documenting cross-cultural differences has accumulated, researchers have begun to explore the origins of cultural differences in attention patterns (see Miyamoto, 2013 for a review). For example, it has been suggested that people who live in communities with ecological and economic factors that require close cooperation in collective tasks are especially likely to show holistic patterns of attention (Uskul et al., 2008; Talhelm et al., 2014). Talhelm and his colleagues found that regional variations in holistic attention in China can be explained by the type of farming prevalent within each area. People who live in regions where rice-farming—an economic activity that requires close cooperation and coordination among members of the community—is prevalent show a more holistic pattern of attention than people who live in wheat-growing regions. Because natural ecological factors that require more communal labor are more common in East Asia than in the West, these findings suggest that the need to engage in collective tasks partly underlies cultural differences in holistic attention.

However, even if there is a correlation between cooperation and holistic attention at the group level, the same correlation does not necessarily exist at the individual level (i.e., the ecological fallacy; Robinson, 1950; Na et al., 2010). In fact, a recent study that manipulated engagement in collective (vs. solitary) tasks failed to find any effects on holistic (vs. analytic) cognition (Magid et al., 2017). Thus, whether and how engagement in collective tasks leads to holistic attention at the individual level remains unclear.

### Holistic cognition in social contexts: social functions and negative affect

To understand the link between holistic cognition and engagement in collective tasks at the individual level, we propose that it is important to take into account the social functions of holistic cognition and its relationship to negative affect that may arise in collective tasks. It has been suggested that holistic cognition serves social functions in collective tasks, namely, facilitating behavioral alignment with others, which helps individuals build, maintain, and restore social harmony (Chartrand and Bargh, 1999). Specifically, van Baaren et al. (2004) found a bidirectional relationship between behavioral mimicry and context-dependent cognition, wherein context-dependent information processing facilitated behavioral mimicry, and vice versa. Thus, holistic cognition may play a role in helping individuals build, maintain and restore social harmony in interpersonal communications.

Such social functions of holistic cognition, especially its relationship-restoring function, may explain why more holistic patterns of cognition are found when people experience a threat to social harmony or negative emotions in social contexts, such as a “fear of isolation” (Kim and Markman, 2006) or *taijin kyofusho* (Norasakkunkit et al., 2012). A study by Kim and Markman (2006) revealed that those who recalled being isolated from others were more likely to exhibit holistic cognition in a memory recognition task than those who recalled having isolated other people (Masuda and Nisbett, 2001). In addition, Norasakkunkit and his colleagues found that *taijin kyofusho* (i.e., other-focused social anxiety) was positively associated with a holistic pattern of attention, as measured by the Framed Line Test (FLT; Kitayama et al., 2003), but the reverse pattern was observed for self-focused social anxiety^1^. These findings suggest that negative emotions associated with the potential for negative evaluation by others are associated with holistic cognition, possibly to restore social harmony.

### Current study

Putting these two lines of work together, we propose that engagement in collective tasks is related to holistic attention, particularly when people experience negative affect related to social concerns. This is because negative affect in such contexts may signal potential threats to social harmony, which then motivates strategies to restore it.

It is important to note that some individuals may experience different types of emotions during collective tasks. Negative affect would be likely to occur during collective tasks when people perceive (either implicitly or explicitly) potential threats to social harmony, or the need to restore it. We predicted that after engaging in a collective task, East Asian participants who experienced an increase in negative affect would exhibit enhanced holistic attention. This is because negative affect experienced during collective tasks in interdependent cultures (e.g., East Asia) is a sign that one has failed in the shared task, and thus possibly disrupted social harmony. In such situations, holistic attention can help restore social harmony by increasing one's awareness of the relationships, which facilitates both adjustment behaviors and social mimicry. Conversely, we predicted that such an association would not be observed when participants perform the task by themselves (i.e., in a non-social context). To test this hypothesis, we had Japanese participants perform a musical duet either with another person (as a collective task) or with a computer (as an individual task), after which we measured their patterns of attention using the FLT (Kitayama et al., 2003), as well as their levels of positive and negative affect before and after the task.

## Materials and method

### Participants

In order to ensure that participants had the basic musical ability necessary to perform the task, we required that they all have extracurricular musical experiences and the ability to read musical scores. One hundred and five Kyoto University students (both graduate and undergraduate) with some degree of musical experience participated in this study^2^. The extent of their musical experience ranged from 1 to 18 years. All participants received the same amount of compensation for participating in the study, regardless of their performance.

### Materials

#### Musical duet task score

**Figure 1:**
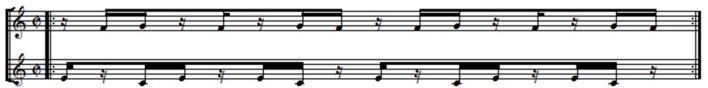
Score used in the Musical Duet Task. The upper musical line is participant A's part, the lower musical line is participant B's part.

An original musical score was created for this research based on the Balinese gamelan style of musical ensemble. This type of ensemble score has two independent parts, which interweave with each other to form a complete rhythmic texture. Mutual coordination between the two musicians is necessary for the notes to alternate accurately. For the entire melody to emerge, participants had to carefully coordinate their performance with that of their partner (or the computer), because most of the musical notes that one person plays must interlock with the notes played by their partner (See Figure 1). Thus, this task requires coordinated collaboration between participants. Participants could easily understand the structure of the duet because each part was simple and consisted of only two notes.

#### Affect before and after the duet tasks

To examine changes in affect during the task, participants were asked to rate their affective state twice: once before and once after the musical task. The questionnaire had four items for positive affect (friendly, happy, proud, and cheerful) and seven items for negative affect (distracted, depressed, angry, sad, frustrated, worried, and embarrassed). These positive and negative items were included in the scale used in Uchida et al. (2008)^3^. Respondents used Likert-type scales ranging from 1 = weakest to 5 = strongest. Mean scores for pre-positive, post-positive, pre-negative, and post-negative emotions were calculated separately.

Cronbach's alpha-coefficients showed good reliability: for positive affect, α = 0.73 (pre-session) and 0.83 (post-session); for negative affect, α = 0.89 (pre-session) and 0.88 (post-session). Changes in positive affect (CPA) and changes in negative affect (CNA) during the duet task were calculated by subtracting pre-session affect scores from the post-session affect scores. For both variables, positive numbers indicate that participants showed increased affect and negative numbers indicate that participants showed decreased affect in the given valence domain during the duet tasks.

#### FLT (Kitayama et al., 2003)

Participants were told that they would perform some simple cognitive tasks. They were given both an absolute task and a relative task, receiving specific instructions directly before performing each task. The order of the tasks was counterbalanced amongst participants. Following the procedure used by Miyamoto and Wilken (2010), for each trial, an experimenter showed the participant a square frame within which a vertical line was printed for 3s. The line extended downward from the center of the upper edge of the square. The participants were then given a second square frame printed on an answer sheet that was either larger than, smaller than, or the same size as the frame the experimenter showed them. The task was to draw a line in the second frame. In the relative task, the participants were asked to draw a line in the response square that had the same proportion to its frame as the line in the original square had to its frame. In the absolute task, participants were asked to draw a line in the response square that was identical in length to the line from the original square. Each task contained one practice trial and five real trials. To minimize error, the experimenter gave feedback if participants drew a line that did not follow the instructions during the practice trial.

To assess performance on the FLT, we calculated the error in each trial as the absolute difference between the length of lines drawn by participants and the correct lengths.

#### Subjective evaluation of the task difficulty

We asked participants in both conditions to rate how difficult the musical duet task was on a Likert-type scale ranging from 1 = very difficult to 4 = very easy. The score was reversed (1 = very easy, 4 = very difficult) so that higher scores indicate greater task difficulty.

### Procedures

An equal number of sessions were run in both conditions: 35 sessions in collective condition and 37 sessions in individual condition. The collective condition included 35 pairs of unacquainted students (28 males and 40 females), and the individual condition included 37 participants (16 males and 20 females, one not disclosed) who worked on their own^4^. Within each pair in the collective condition, gender was matched, and participant age and musical experience were matched as closely as possible^5^.

The procedures used in this work were in accordance with the American Psychological Association Ethical Guidelines and approved by the Kokoro Research Center at Kyoto University following the Japanese Psychological Association guidelines. All participants gave their informed consent and were debriefed and informed about the true purpose of the research immediately after the experiment.

#### Collective condition

Pairs of participants sat at a table with a partition between them to prevent communication. First, participants answered a questionnaire about their current level of negative and positive affect to provide a pre-session baseline of affective state. They were then shown a video clip in which two people correctly played a duet together on xylophones twice. Afterwards, we gave each participant a xylophone and a musical score. The musical score was comprised of two parts (see Figure 1), of which each participant was assigned to play one. First, they practiced their own part individually for 3 min, beginning with a rhythm of 120 beats per minute and gradually increasing the tempo according to their abilities. Participants could play a video clip in which a person correctly played their assigned part when they wanted (personal practice phase). After that, they performed together with their partner for 8 min (duet phase). They were instructed to increase the tempo until they were playing as fast as they could. They then exchanged parts and repeated the personal practice phase (3 min) and duet phase (8 min) again. After the musical performance session, participants answered a questionnaire about their current negative and positive affect to provide a post-session measure of affective state. The participants then performed the FLT (Kitayama et al., 2003). After completing FLT, the participants went to separate rooms where they filled out demographic questionnaires (e.g., age, sex)^6^.

#### Individual condition

The procedure was the same as in the collective condition, but in the individual condition, participants performed the duet with a sound clip from a PC, which would gradually increase the tempo during the performance phase.

## Results

### Subjective evaluation of the task difficulty

An independent-sample *t*-test revealed a significant difference of task difficulty between conditions, *t*_(102)_ = 3.13, *p* = 0.002, [95% confidence interval (CI) = 0.17, 0.77], Hedge's *g* = 0.64. Participants in the individual condition (*M* = 3.22, *SD* = 0.48) reported that the task was significantly more difficult than those in the collective condition (*M* = 2.75, *SD* = 0.84). This is probably because adjusting one's performance to computer playback is relatively more difficult than adjusting to a human partner. To control for the effect of task difficulty, we included task difficulty as a covariate in the main analyses.

### Affect change before and after the duet tasks

We compared the change in positive and negative affect during the duet tasks in the two conditions. Independent-sample *t*-tests showed that there were differences between the two conditions: for positive affect, *t*_(102)_ = 2.91, *p* = 0.004, [95% CI = 0.14, 0.74], Hedge's *g* = 0.49; for negative affect, *t*_(102)_ = 2.41, *p* = 0.02, [95% CI = 0.06, 0.59], Hedge's *g* = 0.59. The mean scores for the change of positive affect (CPA) were 0.09 (*SD* = 0.64) in the collective condition and −0.34 (*SD* = 0.88) in the individual condition; the change of negative affect (CNA): −0.14 (*SD* = 0.51) in collective condition and 0.19 (*SD* = 0.88) in individual condition. In line with the difference in task difficulty, participants in the individual condition were more likely to show a decrease in positive affect and an increase in negative affect than those in the collective condition.

### FLT

We performed a two-way Mixed-Model ANOVA with a condition (between: collective vs. individual) × FLT task type (within: absolute vs. relative) design. The main effect of the FLT task type proved significant, *F*_(1, 103)_ = 29.20, *p* < 0.001, [95% CI = 10.88, 56.04], $\eta_{p}^{2}$ = 0.22). Replicating previous studies on East Asian patterns of attention, Japanese participants were better at the relative task than at the absolute task. The condition did not interact with the FLT task type, *F*_(1, 103)_ = 1.52, *p* = 0.22, [95% CI = 0, 10.23], $\eta_{p}^{2}$ = 0.01, suggesting that, in general, engaging in collective tasks (vs. individual tasks) does not itself influence error score patterns on absolute or relative FLT tasks.

#### Affect change × condition

**Figure 2:**
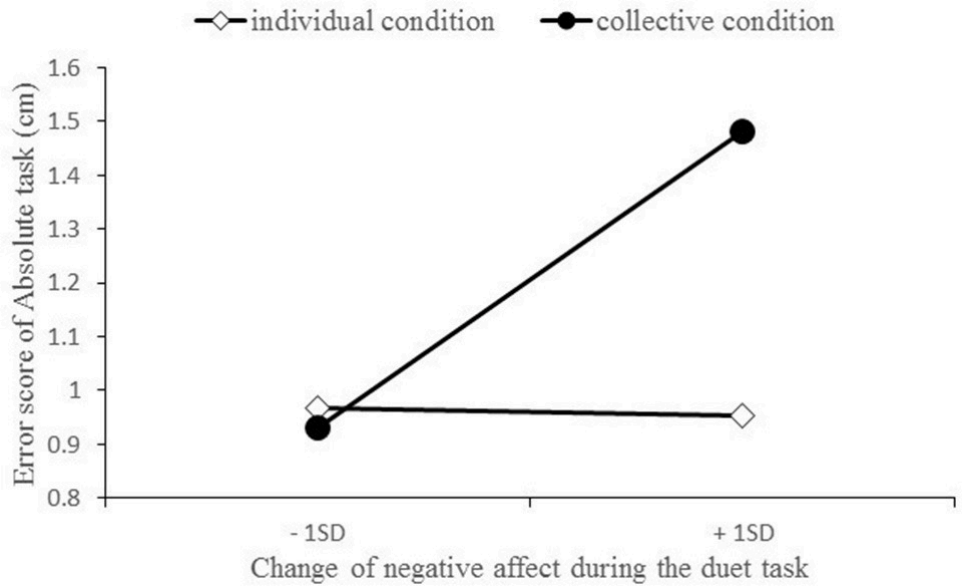
The interaction between negative affect change and condition on the error score of the Absolute task in the FLT. The x-axis indicates the score of the change in negative affect during the duet tasks (plotted at 1 SD below and above the mean). The figure depicts the simple slopes calculated based on multiple regression analyses for the collective condition and individual condition.

We conducted two separate multiple regression analyses on each mean error score of FLT (dependent variable), using dummy-coded conditions (collective condition = 1, individual condition = 0), CNA (change of the negative affect), and CNA x condition interaction as predictors (independent variables), with CPA (change of the positive affect) and task difficulty as control variables. Consistent with our hypothesis, there was a significant CNA × condition interaction on the error score of absolute task *b* = 0.415, *SE* = 0.197, *p* = 0.04, [95% CI = 0.023, 0.807], β = 0.261. CNA positively predicted error scores on the absolute task in the collective condition, *b* = 0.406, *SE* = 0.174, *p* = 0.02, [95% CI = 0.027, 0.686], β = 0.424, but not in the individual condition, *b* = −0.01, *SE* = 0.137, *p* = 0.94, [95% CI = −0.296, 0.217], β = −0.010 (Figure 2). However, the CNA × condition interaction was not significant for the error score of relative task; *b* = 0.089, *SE* = 0.069, *p* = 0.20, [95% CI = −0.048, 0.226], β = 0.165.

We also explored whether a parallel pattern could be found for CPA to predict each error of FLT tasks. We conducted an equivalent multiple regression analysis. The CPA × condition interaction on the error score of absolute task was not significant *b* = −0.279, *SE* = 0.177, *p* = 0.12, [95% CI = −0.629, 0.072], β = −0.224. Although the effect was not significant, the pattern of the interaction was congruent with the interaction observed for CNA^7^. No significant result was found on the error of relative task; CPA × condition interaction: *b* = −0.045, *SE* = 0.062, *p* = 0.47, [95% CI = −0.167, 0.077], β = −0.106. The full results of the multiple regression analyses are summarized in Table 1. Additionally, controlling for age and gender in the analyses did not affect the results^8^.

**Table 1:** Full results of multiple regression analyses.

	**Error score of absolute task**				**Error score of relative task**			
**Predictors**	**β**	***t***	***p-value***	**Fit**	**β**	***t***	***p-value***	**Fit**
Dummy-coded conditions (collective = 1, individual = 0)	0.18	1.72	0.09		0.07	0.66	0.51	
Change in negative affect (CNA)	−0.01	−0.07	0.94		−0.05	−0.31	0.76	
Subjective evaluation of musical task difficulty	−0.02	−0.16	0.87		0.14	1.34	0.18	
Change in positive affect (CPA)	−0.04	−0.32	0.75		0.08	0.59	0.55	
Condition x CNA	**0.26**	**2.10**	**0.04**		0.17	1.30	0.20	
				$R_{adj}^{2}$ = 0.05				$R_{adj}^{2}$ = −0.01
Dummy-coded conditions (collective = 1, individual = 0)	0.18	1.71	0.09		0.07	0.66	0.51	
Change in positive affect (CPA)	0.11	0.67	0.51		0.14	0.85	0.40	
Subjective evaluation of musical task difficulty	0.01	0.09	0.93		0.15	1.42	0.16	
Change in negative affect (CNA)	0.15	1.24	0.22		0.06	0.45	0.66	
Condition × CPA	−0.22	−1.58	0.12		−0.11	−0.73	0.47	
				$R_{adj}^{2}$ = 0.03				$R_{adj}^{2}$ = −0.02

*Bold numbers indicate significant results (p < 0.05)*.

## Discussion

These results indicate that holistic attention is more likely to be observed when participants experience increased negative affect in collective tasks than when they do not. Such effects were not observed in the individual task. These results support our theoretical hypothesis that participants who experience negative affect in a collective task will exhibit enhanced holistic attention. Negative affect in collective tasks may signal a disruption to social harmony that an increase in holistic attention may help to restore by facilitating behavioral adjustment and mimicry.

Our results showed that negative affect experienced in a social context was associated with a holistic pattern of attention, but negative affect experienced in a non-social context was not. At least in the context of a musical performance task, the social context of negative affect seems to moderate the effects of negative affect^9^. This finding points to the importance of examining the specific contexts in which affect occurs to understand their consequences. It also suggests that individual differences in emotional reactions to the social context can moderate how engagement in collective tasks impacts holistic attention. While previous studies have repeatedly shown collective-level differences, such as a greater prevalence of holistic attention in interdependent, collectivistic societies (Kitayama et al., 2009), there is little data regarding individual-level links between holistic attention and interdependence (for an exception, see Kühnen and Oyserman, 2002). The present study provides a potential explanation for this apparent cultural difference: cultural tasks in interdependent societies foster engagement in collective tasks at a societal level. At the same time, there is individual-level diversity in how one reacts to or learns from a task (e.g., holistic attention), and one key factor underlying individual differences is emotion. Specifically, individuals who experience negative emotions in collective tasks are likely to show greater holistic attention than those who do not experience negative emotions. On the other hand, cultural tasks in independent societies are less likely to involve collective tasks. Thus, independent societies may have fewer affordances for holistic attention, as there are fewer opportunities to experience negative affect in collective tasks. Thus, despite the lack of a main effect of engagement in collective tasks on holistic cognition at the individual-level, societal-level differences in the frequency of collective tasks may cause societal-level differences in holistic cognition.

Previous studies have shown that negative affect promotes bottom-up thinking and makes people more accommodating (vs. top-down, assimilative thinking; see Forgas, 2013 for a review). In addition, the broaden-and-build theory of positive affect suggests that positive affect broadens the scope of attention and thought-action repertoires (Fredrickson, 1998, 2001), leading to global cognition (Fredrickson and Branigan, 2005). We believe that our study differs from these theories in two important ways that expand on, rather than contradict, them. First, whereas the previous studies tended to focus on emotions experienced in interpersonally neutral situations, the present research showed that negative affect experienced during collective tasks is linked to holistic cognition. Second, it is important to note that holistic cognitive processing, which is the processing of the relationships between components (e.g., attending to relationships between a focal object and its context rather than focusing on the focal object itself), is orthogonal to top-down and global cognitive processing, which focuses on processing the hierarchically higher (vs. lower) properties of a structure (e.g., attending to a larger triangle rather than to smaller circles that make up that triangle; Miyamoto, 2013). Therefore, our current findings, which suggest that negative emotions (in collective tasks) are linked to attention to relational/contextual information, are independent from the broaden-and-build theory, as this theory posits that positive emotions facilitate attention to hierarchically higher levels of information.

We must acknowledge some limitations in the design of our experiment, as well as point out some possible directions for future research. Firstly, in the current study, the change in negative affect in the collective task was measured rather than manipulated. Thus, there is a possibility of reverse causality or an unknown third variable. For example, it may be possible that individuals with a more holistic style of attention are predisposed to feel more negative affect than those with a more analytic attention style. Also, there is the possibility that decreases in negative affect reduced holistic cognition and increased analytic cognition. Although this reverse causality cannot explain the fact that participants exhibiting holistic attention did not report more negative affect than those exhibiting analytic attention in the individual task, future studies should elucidate the causal relationship between cognitive style and negative affect in collective tasks. In addition, positive and negative affect have various domains (e.g., engaging vs. disengaging/ high arousal vs. low arousal). One possible hypothesis is that, even among negative emotions, engaging negative emotions (e.g., guilt) may be especially likely to enhance holistic attention in a collective task. Since this study only focused on emotional valence, future studies should explore the effect of the other affect domains.

Secondly, future research needs to examine whether enhanced holistic attention after negative affect in collective tasks is actually the result of a motivation to restore relationships, and also whether enhanced holistic attention functions in a way that facilitates social harmony in collective tasks (van Baaren et al., 2004).

Thirdly, the predicted interaction between negative emotion and condition was significant for the absolute task, but not for the relative task. Because the amount of error in the relative task was much smaller than that in the absolute task, there might have been smaller room for situational factors to influence the performance in the relative task. Previous studies examining the effects of situation on the FLT also found effects on the absolute, but not on the relative tasks (Guinote, 2007; Miyamoto and Wilken, 2010). Future studies should confirm the current results by assessing holistic attention using other tasks, such as a memory recognition task (Masuda and Nisbett, 2001).

Lastly, the current study examined only Japanese participants. Because previous studies have shown a relationship-restoring function of holistic attention in both Easterners and Westerners (van Baaren et al., 2004; Kim and Markman, 2006; Norasakkunkit et al., 2012), we believe that the current findings would be found across cultures. However, it is also possible that the effects of negative affect in collective tasks may be stronger in East Asian cultures due to the importance that these cultures place on collective tasks. Future research should examine such possibilities.

In summary, negative affect experienced during collective tasks signals potential threats to social harmony that motivate people to restore relationships with partners by increasing holistic attention. The present study revealed one role that negative affect plays in the relationship between cooperation in collective tasks and holistic patterns of attention. Together with the findings that there are more opportunities for close cooperation in farming communities than other types of communities (Uchida et al., 2015) and that East Asians are more prone to experience social anxiety than European Americans (e.g., Norasakkunkit and Kalick, 2002), this work may bridge the gap between individual- and group-level associations between holistic attention and collective work. Even if holistic attention correlates with cooperation at the group-level, the same correlation does not necessarily exist at the individual-level (Magid et al., 2017). The present research suggests the importance of taking into consideration individuals' subjective experiences while engaging in collective tasks to understand how this leads to holistic attention at the individual-level.

## Ethics statement

This study was carried out in accordance with the recommendations of Ethical Principles of Psychologists, Ethical committee of Japanese Psychological Association with written informed consent from all subjects. All subjects gave written informed consent in accordance with the Declaration of Helsinki. The protocol was approved by Ethical committee of Japanese Psychological Association.

## Author contributions

HT, YU, and YM conceived and designed the experiment; TY composed the music and designed the Musical Duet Task; HT performed the experiment; HT analyzed the data; HT wrote the paper and YU, YM, and TY revised the paper.

### Conflict of interest statement

The authors declare that the research was conducted in the absence of any commercial or financial relationships that could be construed as a potential conflict of interest.

## Footnotes

^1^At the same time, however, Zhou and colleagues showed that a relatively negative internal state (lack of control) experienced during individual tasks leads to analytic attention (Zhou et al., 2012), which implies that negative states do not always lead to holistic attention.

^2^A power analysis indicated that a sample size of 100 participants was required for the study to have a medium expected effect size (α = 0.05, power = 0.80, *f*^2^ = 0.15).

^3^The change scores (before and after the task) of 11 affect items were submitted to a factor analysis to confirm the valence structure of the affective scale. The result indicated that the scale consisted of one factor of valence (Factor 1 explained 31%; Scree plot shows 4.35, 0.73, 0.58, 0.49, 0.38, 0.26, 0.21). All negative affect items loaded positively on the first factor (loadings ranged from 0.36 to 0.75), whereas all positive affect items loaded negatively on the same factor (loadings ranged from −0.57 to −0.36). We thus divided the items into negative affect and positive affect.

^4^Confederates performed the duet task in two of the collective condition sessions because one of the participants did not show up.

^5^Participant demographics for each condition were as follows: the mean age was 19.93 (*SD* = 2.02) in collective condition and 20.42 (*SD* = 1.66) in individual condition; the mean length of musical experience was 11.78 (*SD* = 3.94) years in collective condition and 10.25 (*SD* = 4.33) years in individual condition.

^6^Participants also responded to questions about their reactions to the duet task and participated in an emotion perception task (Masuda et al., 2008). As these measures are not directly relevant to the focus of this paper, they will not be discussed.

^7^The simple slope analysis revealed that CPA negatively predicted participants' holistic patterns of attention in the collective condition *b* = −0.185, *SE* = 0.136, *p* = 0.18, 95% CI [−0.455, 0.085], β = −0.217, but this was not the case in the individual condition *b* = 0.094, *SE* = 0.140, *p* = 0.51, 95% CI [−0.184, 0.372], β = 0.110.

^8^Because age is a salient dimension that influences interpersonal interactions in Japan (Nakane, 1970) and gender influences emotional processing (McClure, 2000; Baron-Cohen et al., 2005), we also controlled for age and gender. The analyses showed that the CNA x condition interaction on the error score of absolute task was significant, *b* = 0.485, *SE* = 0.198, *p* = 0.02, [95% CI = 0.092, 0.879], β = 0.305, and that CPA × condition interaction on the error score of absolute task was marginally significant, *b* = 0.323, *SE* = 0.177, *p* = 0.07, [95% CI = −0.674, 0.029], β = −0.260. Both interactions were consistent with our hypothesis. No significant result was found on the error of the relative task for either type of affect change, CNA × condition interaction: *b* = 0.091, *SE* = 0.070, *p* = 0.20, [95% CI = −0.048, 0.230], β = 0.168; CPA × condition interaction: *b* = −0.046, *SE* = 0.062, *p* = 0.46, [95% CI = −0.170, 0.077], β = −0.109. In addition, we subtracted the mean error score for relative tasks from the mean error score for absolute tasks to assess holistic patterns of attention. We also conducted a multiple regression analysis to predict the computed score of holistic attention. See Appendix in Supplementary Material for detailed results.

^9^Moreover, positive affect in the social (but not in the non-social) context showed a pattern of attention that was the reverse of a holistic pattern, but this was not significant.
