# Supplementary material for: Negative Affect during a Collective (but Not an Individual) Task Is Associated with Holistic Attention in East Asian Cultural Context
Source: Front Psychol. 2017 Aug 4;8:1283. doi: 10.3389/fpsyg.2017.01283 (PMC5543282; doi:10.3389/fpsyg.2017.01283)
Supplement: Supplementary file 1 [file Table1.docx]

**Appendix**

**Multiple regression analyses for Holistic pattern of attention**

To assess holistic patterns of attention, the mean error scores of relative tasks were subtracted from the mean error scores of absolute tasks. A multiple regression analysis using dummy-coded conditions (collective condition = 1, individual condition = 0), CNA, and CNA x condition interaction (independent variable), with PAE and task difficulty as control factors was conducted to predict holistic patterns of attention. Consistent with our hypothesis, the result was marginally significant for CNA x condition interaction *b* = .326, *SE* = .191, *p* = .09, 95% CI [-.053, .705], *β* = .213, which means that CNA positively predicted participants’ holistic pattern of attention in the collective condition, *b* = .331, *SE* = .168, *p* = .05, 95% CI [-.003, .666], *β* = .359, but it was not related to their attention in the individual condition, *b* = .005, *SE* = .132, *p* = .97, 95% CI [-.258, .268], *β* = .006.

We also explored whether a parallel pattern could be found for CPA to predict holistic patterns of attention. A multiple regression analysis using dummy-coded conditions, CPA, and CPA x condition interaction as predictors and CNA and task difficulty as control variables was conducted, but the CPA x condition interaction was not significant *b* = -.234, *SE* = .170, *p* = .17, 95% CI [-.572, .104], *β* = -.196.

**Table S1. Correlation matrix for all measure used in current study**

|  | 1 | 2 | 3 | 4 | 5 | 6 | 7 | 8 | 9 |
| --- | --- | --- | --- | --- | --- | --- | --- | --- | --- |
| 1. Error score of Absolute task |  |  |  |  |  |  |  |  |  |
|  |  |  |  |  |  |  |  |  |  |
| 2. Error score of Relative task | **.28** |  |  |  |  |  |  |  |  |
|  | (.00) |  |  |  |  |  |  |  |  |
| 3. Difference of error between FLT tasks  (Relative - Absolute) | **.94** | -.06 |  |  |  |  |  |  |  |
|  | (.00) | (.53) |  |  |  |  |  |  |  |
| 4. Dummy-coded conditions  (collective = 1, individual = 0) | .14 | .04 | .13 |  |  |  |  |  |  |
|  | (.15) | (.70) | (.18) |  |  |  |  |  |  |
| 5. Change in negative affect during duet task | .13 | .02 | .13 | **-.24** |  |  |  |  |  |
|  | (.19) | (.82) | (.20) | (.02) |  |  |  |  |  |
| 6. Change in positive affect during duet task | -.09 | .04 | -.11 | **.28** | **-.60** |  |  |  |  |
|  | (.36) | (.65) | (.26) | (.00) | (.00) |  |  |  |  |
| 7. Subjective evaluation of duet task difficulty | -.05 | .12 | -.09 | **-.30** | .19 | -.06 |  |  |  |
|  | (.65) | (.21) | (.36) | (.00) | (.06) | (.55) |  |  |  |
| 8. Age | -.10 | -.09 | -.08 | -.12 | .12 | -.15 | .00 |  |  |
|  | (.29) | (.36) | (.44) | (.24) | (.23) | (.12) | (1.00) |  |  |
| 9. Dummy-coded gender (male = 1, female = 0) | .13 | -.09 | .16 | -.03 | .01 | .05 | .02 | **-.21** |  |
|  | (.20) | (.39) | (.10) | (.80) | (.94) | (.60) | (.83) | (.03) |  |
| 10. Length of musical experience (years) | -.09 | -.13 | -.05 | .17 | -.12 | -.08 | **-.36** | .07 | **-.20** |
|  | (.37) | (.18) | (.64) | (.08) | (.22) | (.44) | (.00) | (.49) | (.05) |

*Note. Values in parentheses indicate p-values. Bold numbers indicate significant correlations (p < .05).*
